# BMIVPOT, a Fully Automated Version of the Intravenous Pole: Simulation, Design, and Evaluation

**DOI:** 10.1155/2020/7963497

**Published:** 2020-08-10

**Authors:** Abbas Sayed-Kassem, Nancy Kozah, Georges Hajj-Moussa, Reem Harb, Amira J. Zaylaa

**Affiliations:** ^1^School of Engineering, Lebanese International University, Beqaa, Lebanon; ^2^Galien Medical Services Company, Hazmiyeh, Lebanon; ^3^Department of Electrical and Computer Engineering, Faculty of Engineering, Beirut Arab University, Beirut, Lebanon; ^4^Neuroscience Research Center, Faculty of Medical Sciences, Lebanese University, Beirut, Lebanon; ^5^Faculty of Public Health-I-V, Lebanese University, Beirut, Lebanon

## Abstract

Robotic intravenous poles are automated supportive instrument that needs to be triggered by patients to hold medications and needed supplies. Healthcare engineering of robotic intravenous poles is advancing in order to improve the quality of health services to patients worldwide. Existing intravenous poles in the market were supportive to patients, yet they constrained their movement, consumed the time of both the patient and the nurse, and they were expensive in regard to what they offer. Although robotic poles overcame some of the movement limitations of the commercial/market poles, they were partially automated and did not offer additional technological features. The aim of our work was to develop a fully automated Biomedical Intravenous Pole Robot (BMIVPOT) to resolve the aforementioned limitations and to offer new technological features to intravenous poles, thereby promoting the health services. Several sensors and build-up materials were empirically chosen to be cost-effective and fulfill our needs. The new prototype was divided into three steps: simulated prototype, real implementation of the prototype, and testing and evaluation. Simulation results showed the best qualitative way to fit all the specifications in the robotic system, such as the shape, sensors, and connections in order to provide the proper functionality of the system. Experimental and real results provided the manufactured parts, implemented sensors, and the final robot. Testing the tracking and the flow sensor performances were provided. Evaluation of our Biomedical Intravenous Pole Robot with alternatives showed that our robot outperforms the other poles in many aspects including the features it offers, the percentage of interventions it comprised, the reliability, and cost-effectiveness. The overall percentage of features offered by our Biomedical Intravenous Pole Robot was 60% higher than that offered by peer research poles and 80% higher than that of the market poles. In addition, the average percentage of integration of interventions (architecture, sensor, wireless, tracking, and mechanical) in the Biomedical Intravenous Pole Robot was at least 56% higher than that of the alternative poles. According to the results, Biomedical Intravenous Pole Robot offers a cost-effective price as compared to the others. As a future prospect, we intend to add more features to this prototype in order to enhance it, such as vital signs detection, and improve the tracking system.

## 1. Introduction

**Figure 1:**
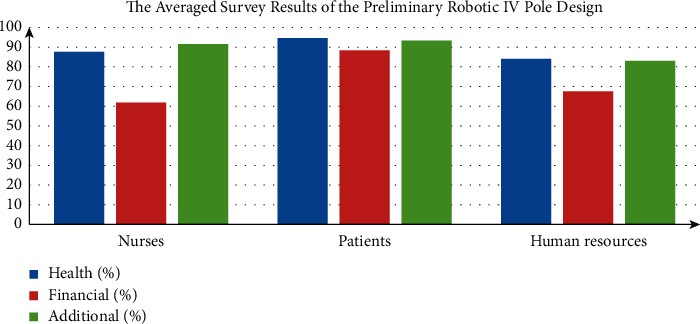
The averaged survey results of the preliminary robotic IV pole design [2].

Robotic intravenous (IV) poles are medical supportive instruments under research that could be partially automated and could hold IV medications to patients in an advanced way [1–3]. With the introduction of research IV poles, such as the so-called autonomous IV poles [1], and the robotic IV pole or novel robotic IV pole [2], some improvement to the commercial/market designs has occurred. The enhancement was centered on the IV poles' field of movement. In the autonomous IV pole designed by Binger et al., the so-called autonomous motion was achieved by the attachment of a nylon twine between the patient and the robotic system [1], while in the preliminary robotic IV pole designed by Sayed-Kassem et al., the automated motion was achieved via a joystick controlled by the patient [2, 3]. According to the survey results showing the benefits of the robotic IV poles from the nurses, patients, and human resources points of view, there was a common agreement on the advantage of the robotic IV pole, which was designed to improve the comfort, cost, and feasibility. The majority of nurses (87.55%), patients (94.60%), and human resources (84%) agreed on the health benefits of the robotic IV pole as shown in Figure 1. Pertaining to the financial benefits of the robotic IV pole, 61.79% of nurses, 88.45% of patients, and 67.50% of human resources agreed on its reasonable price, while the additional benefits exhibited the higher percentage of agreement from patients (93.26%), nurses (91.66%), and human resources (83%), respectively [2].

Despite the survey and the enhancement related to the movement of research IV poles, the motion was not fully automated, and the problems of nurses' time consumption and sensors' lack were not solved completely. By comparing their presented features and their cost, the partially automated IV pole costs around $2000 [1], and the robotic IV pole costs around $300 [2]. Furthermore, the robotic IV poles were considered somehow costly as opposed to market poles [2].

Based on what preceded, the aim of our project was to design a new fully automated prototype for what we named a BioMedical IntraVenous Pole Robot (BMIVPOT). We hypothesize that BMIVPOT enhances the healthcare service to patients in hospitals and medical centers through its new, fully automated, user-friendly, and reasonably priced prototype. Our aim is to (i) improve the tracking system which provides the automated movement of the robot, (ii) enhance the detection of the saline's level, (iii) add new sensors to detect blood leakage in the IV tube, the saline's flow and thus increase the safety of the robot.

Our paper showcased the workflow for building the different parts of the fully automated system. The workflow comprised three main parts: simulation, hardware (combining system parts), and the testing and evaluation steps. The simulation of the system was implemented according to the AutoCAD drawings, which permit the visualization of the different building blocks of the full design. On the other hand, the real construction and hardware included the combination of the materials involved in the development of BMIVPOT. Moreover, the testing and evaluation of the performance and the cost-effectiveness of our prototype was revealed through the graphs and through comparing BMIVPOT to the existing designs and standards.

The remainder of this paper is organized as follows. In Section 2, the types of IV poles existing in the literature were provided. In Section 3, the BMIVPOT's materials and methods were introduced. In Section 4, the prototype's implementation was reported. In Section 5, the three types of results, simulation, real, and testing and evaluation results, were presented. In Section 6, the aforementioned results were discussed, and in Section 7, a general conclusion and future work were provided.

## 2. Existing Robotic Intravenous (IV) Poles

**Figure 2:**
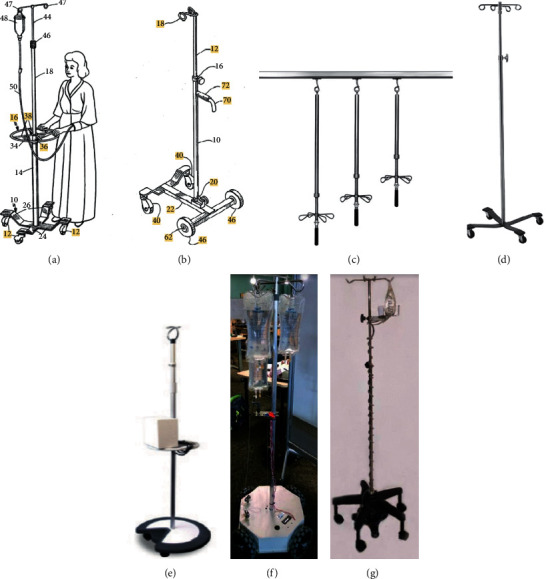
Different intravenous (IV) stands available in the market (top) and in research (bottom). (a) Ambulatory patient support stand [4]. (b) Homecare IV stand [5]. (c) Hanging IV pole [6]. (d) The simple free-standing pole [7]. (e) Dyaun IV stand [9]. (f) Autonomous IV pole [1]. (g) Robotic IV pole [2].

After our thorough review and research on the existing IV poles/stands, these stands can be divided into two main categories: the research IV poles and the IV poles present in the market (shortly market poles). The research IV poles were divided into two, the autonomous IV stand [1] and the robotic IV stand [2], while the market/commercial IV poles were divided into several designs, including, but not limited to, the ambulatory patient support stand [4], Homecare IV stand [5], hanging IV pole [6], Brewer stands [7], and Dyaun IV stand [8, 9] as shown in Figures 2(a)–2(g). The market IV poles are shown in Figures 2(a)–2(e), while research poles are shown in Figures 2(f) and 2(g).

### 2.1. IV Poles in Research

The common research IV pole designs are the autonomous IV stand developed by Binger et al. [1] and the robotic IV stand developed by Sayed-Kassem et al. [2, 3]. These two poles that were used in research are shown in Figures 2(f) and 2(g), respectively.

The autonomous IV stand allows mobile medicine delivery without the need for the patient to maneuver the system [1]. However, in the latter design the patient is tethered to the device via a nylon twine attached to a gait belt that the patient has to wear around the waist. The position of the patient and the angle of measurement are produced by two encoders: the potentiometer and the rotary encoder shaft [1].

Furthermore, the robotic IV pole allows the patient to move the stand using a joystick and releases an alarm whenever the IV bag is emptied [2]. However, the movement of this device depends mainly on the Radio Frequency (RF) communication between the controller (joystick) and the robotic base. This was achieved by sending commands to three Direct Current (DC) motors triggering three omni wheels, thereby controlling the translation and rotation of the robot. The emptiness of the IV bag was detected using a photodiode placed at the lower end of the IV bag [2].

### 2.2. IV Poles in the Market

Market/commercial IV poles are the most common poles used nowadays; they comprise a stand, wheeled base, and hooks assembled side by side, so that the hooks are attached to the top of the stand and the wheeled base is attached to its bottom [4]. These IV poles can be differentiated according to the added features. Ambulatory patient support, shown in Figure 2(a), is a stand associated with a horizontal support handle which aids in moving the stand feasibly. By pushing the pole while holding the handle, the patient exerts less force as opposed to the usual forces applied to push an ordinary IV pole [5] as shown in Figure 2(a). Homecare IV stand, shown in Figure 2(b), is designed by ensuring the center of mass at its base (bottom part), which is attached to two back supportive wheels and two front casters [5]. The latter design provides an easy assembly and disassembly of the device and allows the adjustment of the elevation of the IV bag depending on the patient's height [5]. Moreover, the hanging IV pole, shown in Figure 2(c), is a pole attached to the roof directly above the patient's bed. A drawback of such an IV stand is that it does not permit the patient to maneuver the pole; someone has to always hold the IV bag [6]. Besides, the Brewer stand, shown in Figure 2(d), is a free-standing mobile pole which includes improvement in hangers, accessories, and the adjustment of the stand's height. The primary difference of the Brewer stand as opposed to Figures 2(a)–2(c) is in the design of the base and the number of wheels included. A more advanced free-standing IV pole is the Dyaun IV pole, shown in Figure 2(e); however, it is no longer utilized nowadays [8, 9].

Both the advantages and drawbacks/disadvantages of the IV poles existing in the market and in research were provided in Table 1. The drawbacks reported for the existing poles were centered on its cost, the space it occupies, how it is maneuvered, and the absence of crucial features. Thereby, these reported drawbacks triggered the development of the fully automated BMIVPOT.

## 3. Biomedical Intravenous Pole Robot (BMIVPOT)

**Figure 3:**
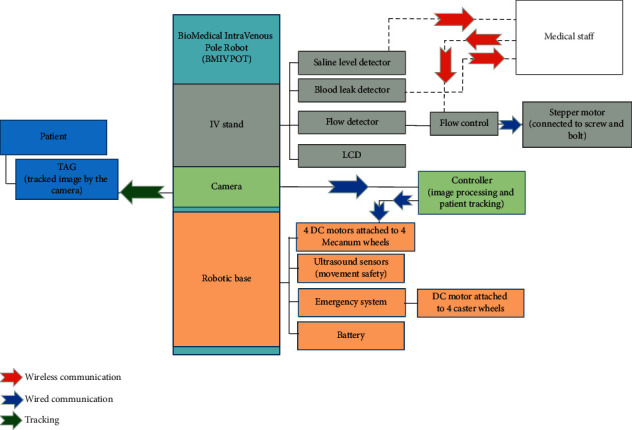
The block diagram of the Biomedical Intravenous Robot (BMIVPOT).

The materials used to develop the novel prototype of the robotic IV pole, i.e., BMIVPOT, were provided. The framework of this design is shown in Figure 3. The tracking system is composed of the camera, the TAG, and the controller of the image processing. The tracking system then drove four DC motors allowing the automated movement of the system. Moreover, the sensors of the saline level and blood leak detectors were implemented on the IV stand, and the parameters measured were displayed on an LCD screen. The measured results were planned to be sent wirelessly to the medical staff allowing them to monitor these parameters and control the flow. In addition to the emergency system, the DC motor was attached to four caster wheels to work traditionally when needed.

### 3.1. BMIVPOT Materials

**Figure 4:**
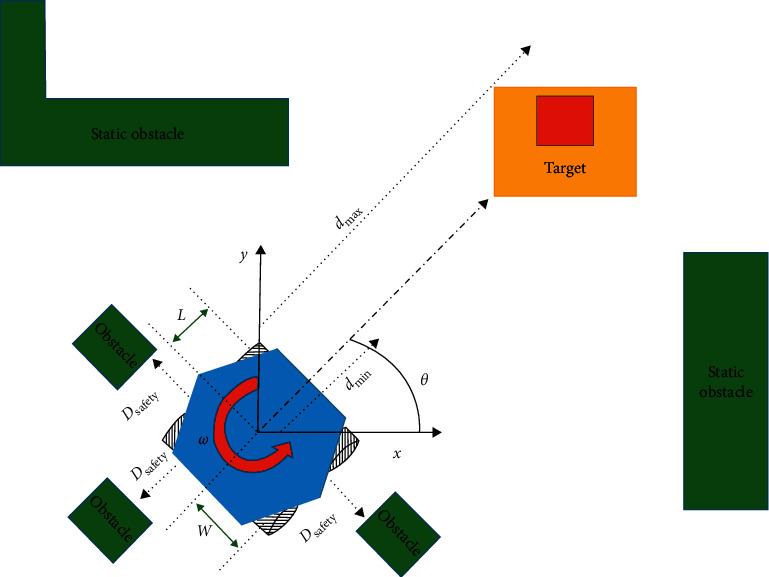
Model of the BMIVPOT, target distance, and TAG.

The materials needed for the implementation of BMIVPOT prototype were selected to achieve the fully automated tracking and to provide new features. Noteworthily, the new features are the detected flow rate, detected blood leak, detected volume, detected obstacle, emergency alarm, linear velocity of BMIVPOT, angular velocity of BMIVPOT, and the distance covered by the BMIVPOT. These materials are listed as follows:

(i) The choice of the architecture materials was based mainly on both the material's weight and availability as compared to several designs. According to Figure 3, the architecture of the BMIVPOT comprised the IV stand, camera, and base. The Plexiglas material was employed due to its common availability, its hygienic property, ease of cleaning, and its cost-effectiveness [10].
(ii) The sensor materials were as follows:
  - A load cell was used to detect the saline's level and thus volume, connected to a load cell amplifier module (HX711) [11] as shown in Figure 3.
  - The blood leak detector and the flowmeter were Infrared Light-Emitting Diode (IR-LED) and photodetector, respectively [12].
  - HC-SR04 ultrasonic sensor was used as an obstacle detector [13, 14].
  - I2C 16 × 2 Arduino Liquid Crystal Display (LCD) module [15] was used to display the different detected and measured parameters.
(iii) The wireless materials included the use of the Wi-Fi NodeMCU shield which was used due to its feasibility and ability to send an analog signal and several digital signals [16, 17].
(iv) The patient's tracking materials were as follows:
  - The Raspberry *Pi* 3 was used due to its capability in running multiple programs simultaneously and performing image processing at a reasonable pace [18, 19].
  - An 8-megapixel camera module V2 was employed due to its compatibility with the Raspberry *Pi* 3 [20]. Thereby, this camera system could recognize the unique TAG, placed on the patient, at larger distances [21], while ensuring the security of each patient through the unique TAG.
  - The TAG used was a square-colored image with a yellow background and red foreground as shown in the model in Figure 4. In order to obtain a unique identification of the patient, the camera was implemented to focus on the aforementioned image. Moreover, each side of the square in the TAG/image was 15 cm. The simple scenario of tracking the target was shown in Figure 4 through a direct straight line. Regardless of the scenario of tracking whether simple or complex, BMIVPOT was planned to maintain a safe distance between its boundaries and any obstacle present within its safety distance (*D*_safety_). Also, the robot was planned to maintain the followed target's center of mass within its Field of View (FOV). BMIVPOT was also planned to maintain the target between the distance *d*_min_ and *d*_max_ in order to keep the target (patient) safe in the presence of static obstacles.
(v) The mechanical materials were as follows:
  - The flow control system was composed of a stepper motor (controlled by a DRV8825 motor driver), screw, and bolt [22]. The stepper motor was controlled by DRV8825 motor driver. The hybrid stepper motor was selected due to its high holding torque and low power consumption [23, 24].
  - Four DC motors were placed in the base and connected to four Mecanum wheels in order to provide a smooth movement associated with all the degrees of freedom [25–27]. The high torque DC geared motor was selected due to its high holding torque and low power consumption [28, 29]. In addition, two relay modules were employed to provide the correct voltage polarity to the DC motors.
  - The Arduino UNO was used to control the DC motors.
  - Another DC motor [28, 30] and four caster wheels were used to form the emergency system.

### 3.2. BMIVPOT Development Method

In order to develop the BMIVPOT, first a simulation of the prototype was carried out using AutoCAD; then the real prototype construction was achieved. The interventions that we have done to provide the new fully automated BMIVPOT were divided into architecture (related to the shape), electronic (choice of sensors), mechanical (flow rate detection), communication (related to the Wi-Fi technology), and the tracking intervention.

#### 3.2.1. Simulation Method

The AutoCAD simulated drawings were used to showcase the architecture intervention revealing the different geometric aspects of the design. The architecture of the prototype was mainly composed of the base and stand. The shape of the base was chosen to be surface efficient; the whole surface was used and filled with electronics. A large surface was required to realize the electronics. Therefore, according to the empirical inference [31], the octagonal shape, having a side of *a*=27.1 cm and an area of $A=2\left(1+\sqrt{2}\right)a^{2}=3540.81\;{\text{cm}}^{2}$ was the best design. This shape solved the trade-off between conserving a large surface and reducing the weight. The BMIVPOT's stand was planned to have a square shape in order to place the required objects on it as shown in the studies of Hajj-Moussa et al. and Kozah et al. [31–33].

Concerning the architecture intervention, it comprised the AutoCAD simulation steps needed to plan for the construction of the real BMIVPOT. The other interventions provided specific functions to the system; the assembly and the connection between all the materials are shown in Figure 3.

#### 3.2.2. Real Prototype Method

The real construction method of BMIVPOT was based on the overall design (architecture), sensors, wireless communication, patient's tracking, mechanical, and healthcare communication services. To achieve the architecture intervention, apply the following:

- Use a total Plexiglas volume of 5.019296 × 10^−3^ · m^3^ and thus a base of overall weight of 5.9 kg according to *W*=*ρV*, with *ρ*_plexi_ = 1180 kg/m^3^. Choose the stand to be aluminum with thin thickness.

Regarding the sensor's consideration,

- Measure the saline's weight to calculate the volume.
- Detect the saline's droplet using the flow meter.
- Count the droplet when it passes between the IR emitter and the receiver, through calculating the change in intensity values detected by the receiver.
- Compute the flow rate by considering the number of drops per minute.
- Place the tube near the IV insertion in the blood detector system, in order to detect any backflow of blood from the patient's vein to the tube.
- Detect the blood leakage when the intensity drop is recorded by the phototransistor, give an alarm, and send a notification to the nurse.
- Place the ultrasound (US) sensors on the four sides of the base in order to detect at least obstacles at 10 cm away from the base and to provide more security to the system.
- Display the parameters measured (flow volume, time) on an LCD screen connected to the main controller.

Regarding the wireless intervention,

- Integrate Wi-Fi shields on the volume detector, the flow meter, the blood leak detector, and the flow control system.
- Provide the detected and calculated parameters (the volume and the flow of the saline) and the blood leakage on the cellphone of the nurses. Thereby, they could monitor and control the saline's flow.

**Figure 5:**
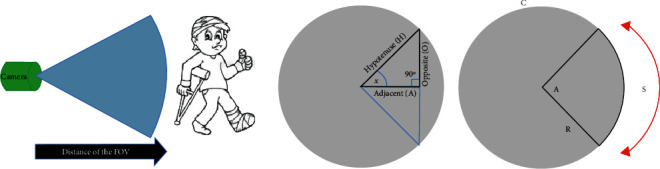
The patient's tracking method from the Field of View (FOV) showing the distance of pixels/m to the distance calculation.

Pertaining to the patient's tracking,

(i) Attach the chosen camera to the IV pole in order to track the patient's movement by TAG recognition. Recall that the TAG was a simple small image printed on the back of the patient's costume. The TAG has the shape of a square of side (*b* = 15 cm).
(ii) Take into consideration that the distance interval has to be maintained between the TAG and the camera based on the forthcoming calculations:
  (a) Identify the TAG by taking 492 pixels/m as an assumption. This resolution was used for the identification of unknown faces in forensic applications.
  (b) Calculate the FOV of the camera according to the study of Hajj-Moussa et al. [31].
  (c) Calculate the maximum distance to get the FOV through the right triangle shown in Figure 5. The opposite side of the right triangle is FOV/2, and the angle (*X*) is half of the angle of the camera's lens. Thereby, the hypotenuse (*H*) is the distance taken from the camera's lens in order to ensure that the desired FOV is equal to 6.4533 m, where the desired horizontal FOV value was 6.67 m. The calculation was based on Figure 5 and the following equations:
    (1) $$\begin{array}{c} \text{Radius}:R=\frac{C}{2\pi }, \end{array}$$
  where *C* is the circumference of the circle.
    (2) $$\begin{array}{c} \text{Circumference}:C=S\left(360A\right), \end{array}$$
  where *S* was the approximated segment of the circle, *R* was the radius (the FOV), and *A* was the angle.
  (d) Concerning the vertical FOV, repeat the same calculation by setting FOV at 5 m, the radius at 5.87, and the distance at 6 m, based on the lens's vertical angle. Thereby, the maximum distance that the 8 MP camera can identify an object was at 6.67 m along the horizontal direction and 5.87 m along the vertical direction, with 492 pixels/m, which was sufficient for the TAG identification [31].
(iii) Process using the Raspberry Pi 3 the captured images in order to identify the patient's position, send the coordinates of the patient to the Arduino UNO, and trigger the IV pole to follow the patient.

Regarding the mechanical intervention,

- Introduce and place a flow control system on the new BMIVPOT. While the motor rotates, the screw rotates performing a translational motion. This final motion causes a pressure on the IV tube which controls the saline's flow.
- Use four DC motors to move the base.
- Assume that the average acceleration of a walking human is 0.65 m/s^2^, and the total mass of the system is approximately 40 kg, including an average additional load that can be attached to the system. The summation of forces has to be equal to the driving force which was 26 N. Thus, according to torque equation *τ* = (force) (radius of wheel), the total torque that the four motors have to handle was 1.3 N · m, i.e., 0.325 N · m for each motor.
- Code the Arduino UNO to control the two relay modules, which then controls the DC motors.
- Drive the DC motor by two relay modules, in order to lift up the casters so that the system moves on the Mecanum wheels, or lower the caster down in case of emergency.

#### 3.2.3. Testing and Evaluation Method

The testing of the system's performance, feature monitoring, and cost effectiveness was based on the accuracy and repeatability of the measurements. While the evaluation was based on comparing the results obtained from BMIVPOT to those existing in the market and in research. To check the function of each sensor, follow the upcoming steps:

- Test the volume meter by applying a series of known-weight stuff and checking if the measured values obtained by the balance were equal to them.
- Test the flow meter by placing the drip chamber of the IV bag in the flow meter's housing and setting the saline at “slow flow” so that you could count the drops manually and check if the flow meter had counted the same number of drops.
- Test the blood leak detector by checking if the alarm is triggered when blood passes in front of the LED and the phototransistor.
- Test the flow control system by checking if the screw was pushing on the tube with the correct pressure to provide the intended flow rate.
- Test the LCD display to verify if the values displayed are the real ones.
- Test the camera and TAG-recognition/tracking of the TAG by checking if the tracking image displayed the correct TAG and contour, also by checking the processing of the images for each frame. Test the TAG-recognition/tracking according to the schematic representation illustrated in Figure 4.
- Test the movement of the system relative to the TAG by measuring the linear and angular velocities and the distance covered by BMIVPOT relative to time.
- Test the wireless communication by verifying the establishment of the connection between the server and the sensor and by verifying the display of results on the server, i.e., whether they were consistent with the measured ones or not.

### 3.3. BMIVPOT Implementation

This section includes the implementation process to build the BMIVPOT. It shows the base implementation, stand implementation, sensors' placement, and tracking system implementation.

#### 3.3.1. Base Implementation

In order to build the base according to the simulated dimensions and overall design, the different parts forming the base were manufactured independently then reassembled. In order to mimic the assembly, apply the following steps:

- Assemble the DC motors to the Mecanum wheels, by inserting the motors' shafts each to a bearing fixed in the wheels.
- Fix the four assembled motor-wheel parts to their specific position on the Plexiglas floor.
- Connect the motors to their relays and to the Arduino Uno, which has to be connected to Raspberry Pi 3 via USB cable.
- Attach the casters to their manufactured holders.
- Fix the motor, responsible for lifting the emergency casters up and down.
- Fit the casters-holder assembly on the floor part, where their holes have to be drilled to provide an easy upward and downward movement for the casters.
- Connect the casters' motor to its driver and to the Arduino and place the batteries inside the base then launch the stand implementation.

#### 3.3.2. Stand Implementation

In order to assemble the stand, apply the following steps:

- Fix the stand to the holder.
- Insert the pyramid-shaped part into the stand then fix it to the floor-casters assembly.
- Fit the flow control stepper motor inside its box and place it on the stand.
- Connect the flow control stepper motor to its driver and to the NodeMCU.
- Attach the hooks at the top of the stand then place the sensors.

#### 3.3.3. Placement of Sensors

In order to achieve the intended functionality of the sensors, place a specific setup suitable for their measured parameters and apply the following steps:

- Connect the load cell to the HX711 module and then to a NodeMCU.
- Fit the load cell circuit inside its box placed under the hooks.
- Place the IR LED and phototransistor inside a black box, where the LED and the phototransistor have to be at the same level and facing each other.
- Connect the LED and the photodetector to a NodeMCU. Then connect a 10 kΩ resistor to the analog reading coming from the phototransistor to the microcontroller pin.
- Place the three US sensors on the three sides of the base and connect them to the Arduino UNO.
- Place the US sensor at the front side of the stand above the camera and connect it to the Raspberry Pi 3.
- Connect the blood leak detector to the NodeMCU and place its circuit on the IV tube cable; then start with implementing the tracking system.

#### 3.3.4. Tracking System Implementation

To synchronize the overall movement of the BMIVPOT with the movement of the patient, apply the following steps:

- Place the TAG on the back of the patient's costume.
- Place the camera on the stand on the predefined height to track the TAG.
- Connect the camera to the Raspberry Pi 3 in order to recognize the TAG and provide image processing.

## 4. Results of BMIVPOT

Herein, the results of BMIVPOT are provided. The results were divided into three parts, the simulation results in Section 4.1, the real results in Section 4.2, and the testing and evaluation results in Section 4.3. As for the testing and evaluation part, it included a comparison between BMIVPOT and existing poles based on the performance, the features it provides, and the cost.

### 4.1. Simulation Results

The AutoCAD simulation results of the different parts of the BMIVPOT were realized and were represented depending on the type of intervention.

**Figure 6:**
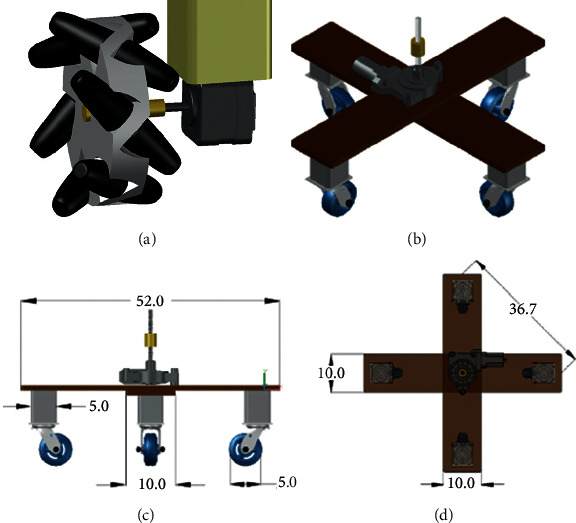
AutoCAD drawings of the BMIVPOT wheels. (a) Mecanum motor simulation. (b) 3‐Dimensional view of emergency system simulation. (c) Side view of emergency system simulation. (d) Top view of emergency system simulation.

The simulated Mecanum wheels and their motors are shown in Figure 6(a), and the simulated emergency system is shown in Figures 6(b)–6(d).

**Figure 7:**
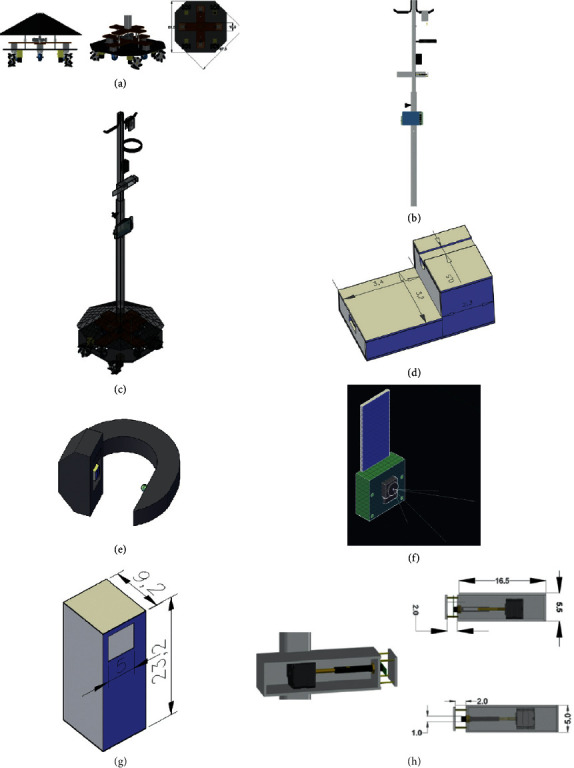
AutoCAD drawings of the BMIVPOTparts. (a) BMIVPOT base. (b) The pole. (c) Whole system. (d) Blood leak detector housing.(e) Flowmeter housing. (f) Camera and its field of view. (g) Raspberry Pi 3 and camera housing. (h) The flow control system.

The architecture intervention was reflected by the simulated results of the system shown in Figures 7(a) and 7(b) which comprises the base and the pole. The base, shown in Figure 7(a), was divided into three parts: the floor, the casters' holder, and the stabilizing part (with three different views). The overall simulated system is shown in Figure 7(c).

The sensor's intervention was reflected by the housing for each sensor is simulated in order to accommodate for the electronics and materials required. The dimension of each box was chosen according to the size of the chosen electronics and the place they occupied. The simulated blood leak detector's box is shown in Figure 7(d). Moreover, the simulated flowmeter's housing is shown in Figure 7(e).

The tracking intervention was reflected by the simulated result of the camera and its FOV as shown in Figure 7(f). The simulated box that fits the camera and the circuitry is shown in Figure 7(g) in real dimensions. Furthermore, the mechanical intervention included the simulated flow control system shown in Figure 7(h).

### 4.2. Real Results

**Figure 8:**
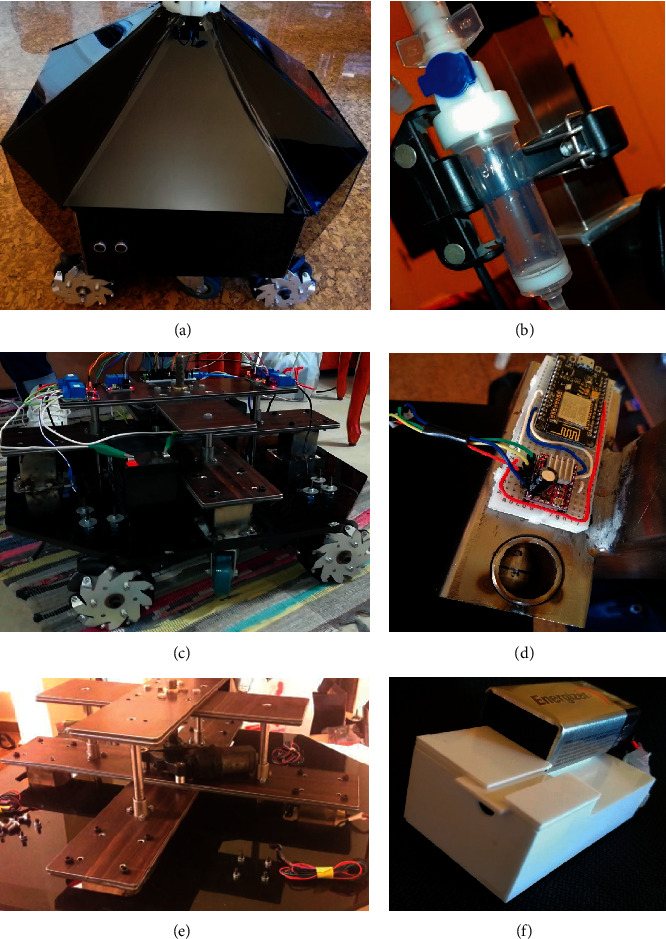
The real BMIVPOT construction results. (a) Whole base assembly. (b) The drip chamber inserted in the flow meter's housing. (c) The emergency system. (d) The flow control system. (e) The casters' holder system. (f) The blood leak detector.

After providing the simulation results of the BMIVPOT, herein the real construction results are provided as shown in Figure 8.

#### 4.2.1. Results according to the Type of Intervention

The architecture intervention results revealed the base of BMIVPOT, the whole base which was assembled as shown in Figure 8(a). The flow meter's electronics were fitted in their housing as shown in Figure 8(b). Moreover, the emergency system shown in Figure 8(c) included the lifting and lowering of the casters, so that the system can move on Mecanum wheels or on the casters, respectively. Besides, the mechanical intervention included the flow control, the motors and wheels, and the emergency system. The flow control system's stepper motor shown in Figure 8(d) was fitted inside the aluminum box and its circuitry was placed on this box. This base was made of three parts as shown in Figure 8(e), with the attached rods and with the casters attached to the system.

Furthermore, the sensors' intervention was reflected by the placement of the blood detector's components inside the Plexiglas box as shown in Figure 8(f).

**Figure 9:**
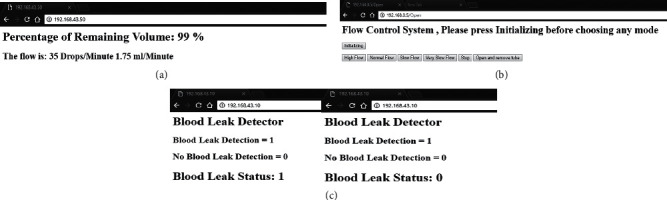
The web server results. (a) Web server showing the volume-flow detection. (b) Flow control server page. (c) Blood leak web server.

The Wi-Fi intervention results showed the communication between the volume detector and the nurse's workstation through the web server and the nurse's workstation (see Figure 9). The flow meter percentages and rates were represented in Figure 9(a). As for the flow control, the wireless communication provided the nurse with the ability to control the flow from the workstation as shown in Figure 9(b). The blood leak detector sent wirelessly a notification to the nurse about any possible blood backflow in the tube as shown in Figure 9(c).

**Figure 10:**
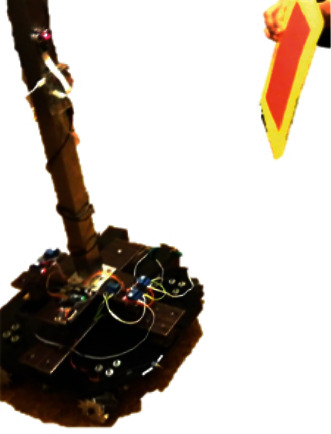
The Biomedical Intravenous Robot (BMIVPOT) tracking the TAG.

The tracking intervention result is shown in Figure 10, where the BMIVPOT was tracking the colored TAG placed on the patient in front of the camera.

### 4.3. Testing and Evaluation Results

**Figure 11:**
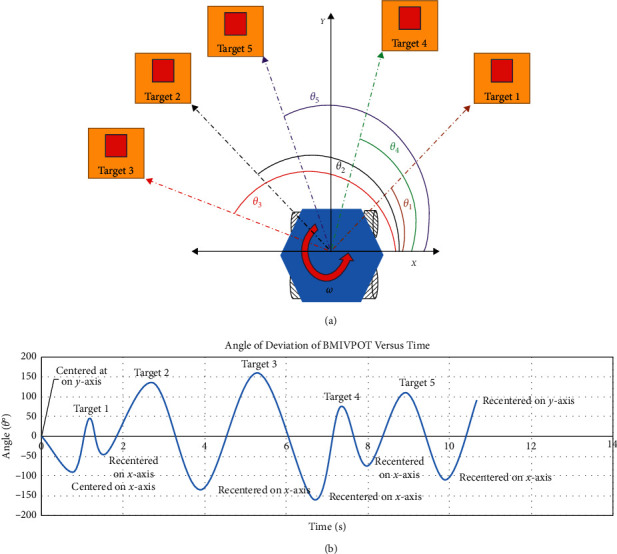
The variation of the movement of the BMIVPOT as a function of the target location and time. (a) The model of testing. (b) The overall profile of the variation of the angle of deviation of BMIVPOT relative to time and target.

The results for testing BMIVPOT tracking system are shown in Figure 11. Noteworthily, the robot could be centered in many ways; in the scenario shown in Figure 11, the center was set to be at the origin of the *x*-axis of BMIVPOT, and the five random targets and angles are shown in Figure 11(a). The results of tracking the TAG at Θ_1−5_ with respect to the *x*-axis are shown in Figure 11(b). The robot was tracking the target while staying centered. The change in the location of the target, i.e., the position of the patient, caused the BMIVPOT to deviate, and the deviation was associated with a change in the angle of movement accordingly. Thereby, the variation of the movement (tracking) of the BMIVPOT was tested as a function of the target location and time. The overall profile of the variation of the movement of the BMIVPOT as a function of the target location and time was aperiodic (see Figure 11(b)).

The positive sign of rotation was chosen to be the counterclockwise direction. Noteworthily, when the target was placed at 45° from the center of the BMIVPOT, the fully automated robot which was centered on *y*-axis, as shown in Figures 11(a) and 11(b), was able to detect the target, rotated 45°, and track the target in 1.2 seconds, merely.

As the target deviates at larger angles, such as Θ_2_ and Θ_3_, the tracking time increases from 2.2 seconds to 3.2 seconds, respectively, by an increment of 1. Thereby, it can be inferred that according to our test when Θ_*i*+1_=Θ_*i*_+Θ′, *t*_*i*+1_=*t*_*i*_+*k*, where *k* = 1 s.

**Figure 12:**
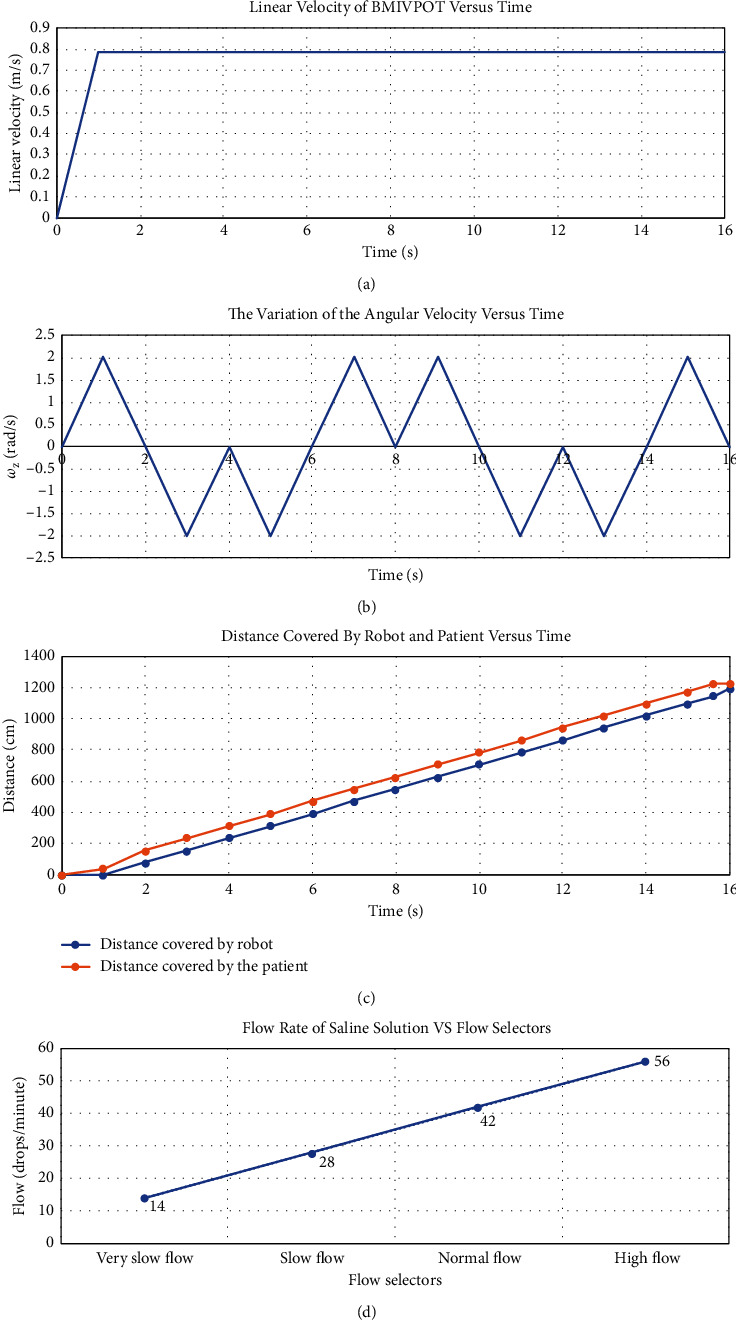
Testing results. (a) The linear velocity of BMIVPOT versus time. (b) The variation of the angular velocity versus time. (c) Distance covered by robot and patient versus time. (d) Flow rate of saline solution versus flow selectors.

Furthermore, the results of testing the movement of BMIVPOT, i.e., the linear velocity, angular velocity, and distance relative to time features, are provided in Figures 12(a)–12(c), respectively. The graph of the variation of the linear velocity of robot versus time (Figure 12(a)) shows that at time *t* = 0 seconds, the robot was at rest; as time increases, the velocity increases gradually to reach to its highest value of 0.785 m/s at *t* = 1 sec. After 1 s, the robot moves at a constant velocity until *t* = 15 s when the patient stopped walking, so its velocity dropped to 0 m/s, while BMIVPOT kept walking with its constant velocity until *t* = 15.618 s to achieve the minimum distance with respect to the patient.

Besides, the graph shown in Figure 12(b) reveals the variation of angular velocity (rad/s) of robot versus time (s). The robot searches for a target by turning left, then returning back to its original position and then turning right. At *t* = 0, the robot is in its original position, where Wz value is 0 (rad/s). When the robot turns left, the angular velocity increases to reach 2.0138 (rad/s) at *t* = 1 s. Noteworthily, the angular velocity was calculated as in the study of Kim et al. [34]. At *t* = 1 s, the robot stops, so the value of Wz decreases back to 0 (rad/s) at *t* = 2 s. Then, the robot starts turning back to its original position at *t* = 2 s where Wz decreases to reach −2.0138 (rad/s) at *t* = 3 s since the moving direction is clockwise. At *t* = 3 s, the robot stops, so Wz increases to reach 0 (rad/s) at *t* = 4 s. At *t* = 4 s, the robot starts turning right, so Wz value decreases to reach −2.0138 (rad/s) at *t* = 5 s. BMIVPOT then stops at *t* = 5 s, so Wz value increases to reach 0 (rad/s) at *t* = 6 s. The robot then starts returning back to its original position, so Wz value increases to reach 2.0138 (rad/s) at *t* = 7 s. Then, the robot stops for 1 s at *t* = 7 s, so Wz values decrease to reach 0 (rad/s) at *t* = 8 s. Then, from *t* = 8 s to *t* = 16 s, the robot repeats the same rotation.

Moreover, the graph shown in Figure 12(c) reveals the distance (cm) covered by the BMIVPOT and patient versus time (s). At *t* = 0 s, both the patient and BMIVPOT were at the initial position close to each other, and the patient accelerates at 0.3925 (m/s^2^). At *t* = 1 s, the distance covered by the robot remains at 0 cm, while the distance covered by the patient increases to reach 39.25 cm, i.e., greater than the minimum distance to keep between the robot and patient, so BMIVPOT starts moving uniformly and following the patient. At *t* = 2 s, the distance covered by the robot increases to reach 78.5 cm, and the distance covered by the patient increases to reach 157 cm. Both covered distances increase until *t* = 15.618 s, where the patient stops walking at a distance of 1147.5 cm, while BMIVPOT keeps moving to achieve the minimum distance *d*_min_ until *t* = 16 s where the distance covered by the patient remains constant at 1226 cm and the distance covered by the robot increases to reach 1196 cm. At *t* = 16 s, the robot stops moving since the distance between the robot and patient becomes 30 cm, i.e., roughly the minimum distance to keep between.

The graph, however, shown in Figure 12(d) reveals the flow rate versus the flow selectors. There were four selectors for indicating the flow of saline infusion. The flow at selector “very slow flow” is 14 drops/minute, so that the time to finish the saline will be 24 hours. The second selector “slow flow” will increase the flow to 28 drops/minute and decrease the time to finish the infusion of saline to 12 hours. Selecting “normal flow” will increase the flow to 42 drops/minutes and decrease the time to finish the saline to 8 hours. At selector “high flow,” the flow will be 56 drops/minute, and the time to finish the saline infusion will be 4 hours. Besides, the results of testing the flow sensors are provided in Figure 12(d).

**Figure 13:**
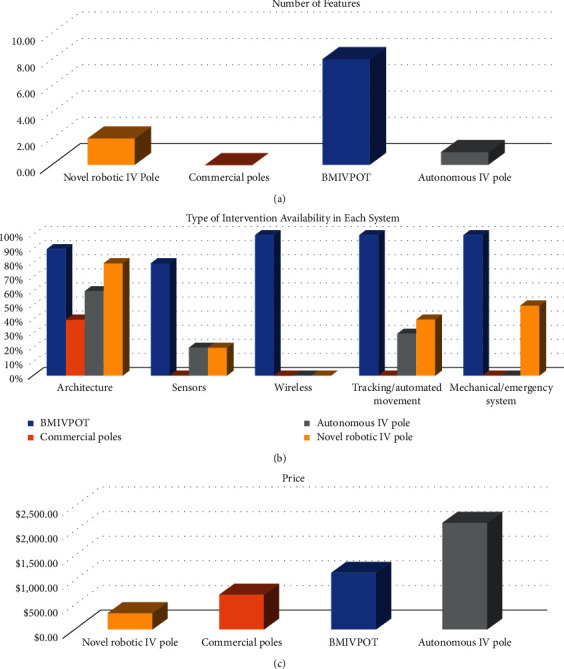
Comparison between different intravenous (IV) pole systems. (a) The number of features in each system in the novel robotic IV pole, commercial poles, BMIVPOT, and autonomous pole. (b) Interventions' availability in each pole. (c) The cost of the poles.

To evaluate our BMIVPOT, the comparison shown in Figure 13 was carried out between the BMIVPOT and the systems existing in the literature, including the novel robotic IV pole proposed by Sayed-Kassem et al., the autonomous IV pole proposed by Binger et al., and other market or commercial IV poles [1, 2].

A comparison perspective was taken into consideration concerning the number of features as shown in Figure 13(a). Noteworthily, all the features taken into consideration were the volume detection, flow rate detection, blood leak detection, flow control feature, wireless communication, emergency system, and the fully automated movement and tracking.

Furthermore, the comparison shown in Figure 13(b) provides the intervention's availability in the BMIVPOT, commercial, autonomous, and the robotic IV poles.

Concerning the architecture, the commercial IV poles were the reference and showed the lowest percentage of 40% (balanced base-pole and hooks design), while the BMIVPOT showed the highest rate of 90% (balanced base-pole with specific built-in material, practical pole design, Mecanum wheels, user-friendly architecture, and lack of hooks' design).

Also for the electronic intervention reflected by the use of sensors, the commercial IV poles in the market showed the lowest percentage of 0% (absence of sensors), while the BMIVPOT showed the highest number of features of 80% (as it possessed 8 features out of 10: obstacle detector, volume detector, blood leak detector, flow meter, emergency alarm, linear velocity detector, angular velocity detector, and distance covered detector and lack of vital signs' sensors, i.e., temperature and blood pressure).

For the wireless intervention, BMIVPOT was exclusively the only system that had a wireless contribution feature (100%). As for the tracking, the BMIVPOT had the maximum percentage of 100% (fully automated), while the novel robotic IV pole had approximately half of the BMIVPOT's percentage 40% (semi-automated and requires prior training).

Concerning the mechanical or emergency system included in each design, the BMIVPOT took the highest rate 100% (since it is fully automated), while the robotic IV pole took the half of this rate (50%: semi-automated).

The evaluation of the price of BMIVPOT was compared to the IV poles designs as shown in Figure 13(c). According to our statistics, the market price of BMIVPOT is $1,155.29 and provided all the eight aforementioned features.

## 5. Discussion

According to our statistics and our thorough literature review, the BMIVPOT which costs $1,155.29 had all the eight aforementioned features. This could be considered as an advantage over the other systems that could cost more or less than the BMIVPOT, when these systems were not capable of providing the same number of features as BMIVPOT. For instance, Sayed-Kassem et al.'s system costs $4325.36 and offers two features [2] as shown in Figures 13(a)–13(c); the cost of each feature was approximately $162.68. Although the cost of our BMIVPOT system is $1,155.29, we had seven features; hence, the cost of each feature was approximately in accordance to that for the novel robotic IV pole $165. As for the autonomous IV pole, it costs $2,167 and provides only one feature [1]. Thereby, our system is considered to be more cost-effective than the two existing alternatives.

Concerning the number of monitored and available features, our system provides seven features (considers 100% from the whole number of features) which surpasses the features monitored and available in other techniques as shown in Figure 13(a). While our system provides seven features, the novel robotic IV pole provides only two features (28.57% from the whole number of features), which are the semi-automated movement and the volume detection. On the other hand, the autonomous IV pole only includes one single feature, which is the automated movement (14.2% from the whole number of features).

In addition to what preceded, by comparing the accuracy of the volume measurement provided by the BMIVPOT to that of the novel robotic IV pole, the volume detection provided by the latter is limited to one volume level, which is the bag emptiness, while the detection provided by our BMIVPOT is based on a continuous volume monitoring for all levels represented by percentage value as shown in Figure 9(a).

Pertaining to the patient's safety and risk management, the BMIVPOT included US sensors which does not harm the patient; this safety measure was absent in the two other systems [1, 2]. According to Sayed-Kassem et al., the patient's safety was provided by the limited range of RF signals sent from the joystick to the pole, which constrain the distance of the patient from the pole [2], thereby giving safety to the IV tube, but does not take into consideration the patient's safety while present in the intended range of motion. Consequently, the BMIVPOT surpasses the novel robotic IV pole and the autonomous IV pole, in the safety and risk management. In addition, the US sensors provide safety for the system itself by keeping it away from obstacles.

By comparing our system to the ambulatory IV pole [4], the Homecare IV Pole [5], and the simple free-standing stands [7], our system included all interventions, while only one intervention, which is the architecture, was present in the commercial IV poles as shown in Figure 13(b). Concerning the architecture intervention, our system was similar in terms of stability, balance, and the overall concept (wheeled base and hooks) to the commercial IV poles [4, 5, 7]. However, BMIVPOT was not similar to the hanging IV pole [6], as the latter pole is attached to the roof merely. Concerning the number of monitored and available features, our system provides all advanced features as opposed to the commercial IV poles as shown in Figure 13(a). Although, commercial IV poles lacked most features, their average cost was above $500, which is considered expensive as shown in Figure 13(c).

The development of BMIVPOT was innovative and research oriented to provide a fully automated IV pole that tracks the patient while moving in a hospital. Several simulations were done in order to provide the appropriate system's architecture and design. Results showed that our BMIVPOT was user-friendly. The user-friendly notation was based on whether the patient and nurse fully trigger the IV pole by themselves, whether they partially trigger the IV pole [1, 2] or whether they do not need to trigger the IV pole (full automation). Thereby, the user-friendly BMIVPOT was associated with the fact that patient/nurse does not need any more to spend effort and time maneuvering the pole as opposed to the existing poles [1, 2]. The BMIVPOT system comprised various interventions, each providing a specific function. BMIVPOT showed up and provided higher performance percentage (80%) as compared to the other existing systems.

The fully automated robotic IV pole, BMIVPOT, exhibited an outstanding progress as compared to the commercial/market IV poles [4–7, 9]. The evolution of BMIVPOT was achieved by transforming the wheeled stand to a robot ready to be used in healthcare organizations. Moreover, the wireless communication provided transfer of eight features to the nurses' workbench. Although the patient tracking seemed to increase the on-chip computation cost, however, each pole followed a unique TAG printed on the back of the patient's costume. Upon testing the aforementioned tracking, the camera was successfully recognizing the tag and following it in the presence of obstacles, and at different angles as opposed to the limitations present in research poles [1, 2].

Furthermore, by comparing our system to the existing research poles, i.e., the autonomous IV pole developed by Binger et al. [1] and the novel robotic IV pole developed by Sayed-Kassem et al. [2], we found that our system surpassed these designs in vast ways, including the number of features, the cost-effectiveness, and the accuracy of the sensors' measurements.

According to Sayed-Kassem et al., the so-called novel robotic IV pole offered patients the ability to maneuver the poles with a joystick, making them move more freely [2], and according to Binger et al., their so-called autonomous system was able to follow the patient, by tethering the patient with a nylon twine to encoders and potentiometers present on the IV pole, which were able to determine the movement and direction of the patient while walking [1]. In comparison to what preceded, our system took into consideration fully automated tracking, movement, and recognition of the patient (100%); however, the motion of the IV pole in the Sayed-Kassem et al. system was semi-automated (50%), since the patient still needs to hold and control the pole by a joystick as shown in Figure 13(b). Moreover, the motion in the Binger et al. system could not be considered automated since the patient is still tethered to the pole, which presents a major limitation on his movement [1].

On the other hand, our BMIVPOT provides a fully automated movement (100%) of the IV pole, because it requires nothing from the patient except to be in the FOV of the camera. The overall percentage of features offered by our Biomedical Intravenous Pole Robot was 60% (80%−20%) higher than that offered by peer research poles and 80% (80%−0%) higher than the market poles. In addition, the average percentage of integration of interventions (architecture, sensor, wireless, tracking, and mechanical) in the Biomedical Intravenous Pole Robot was at least 56% (average interventions of BMIVPOT 94%-average interventions of Novel Robotic IV pole 38%) higher than the alternative poles.

## 6. Conclusion

This work was conducted in order to introduce a new and advanced version of the robotic IV pole, the fully automated BMIVPOT. BMIVPOT is an enhancement of the previous robotic IV pole systems in all terms, including the ease of motion of the patient, the reduced effort spent by the nurses to monitor the IV bag, and other vital signs.

An automated movement was provided and tested to make the patient move freely without the need for dragging his pole and the integration of monitoring sensors to help the nurses. The BMIVPOT was designed to include the tracking of the patient by camera which provides the ability to follow the patient by a fully automated motion.

Furthermore, a wireless communication between the sensors present on the BMIVPOT and their workstation was established, allowing the nurse to monitor the flow, volume and blood leakage wirelessly through the mobile phone. Also, the BMIVPOT has an electronic emergency system, which allows the switching between moving the system either manually on the casters or via the automated motion provided by the Mecanum wheels.

The BMIVPOT could improve the health outcomes for the patients and help the nurses to accomplish their duties and monitor the IV bag.

## 7. Future Work

The BMIVPOT offers many improvements; it could be subject to several enhancements concerning its tracking system, automated movement, the automation of the flow control, and the addition of new biomedical sensors.

The future work emerging from the BMIVPOT construction could be listed as follows:

- Utilizing a more powerful microprocessor able to perform image processing at a higher speed as opposed to the Raspberry Pi 3, also utilizing a specialized tracking camera to reduce the on-chip computation cost
- Improving the overall architecture by making the base smaller
- Utilizing motors and wheels with less noise
- Controlling the automated flow control based on the communication between the volume detector and the flow meter
- Adding new sensors that provide measurements of the vital signs, such as the heart rate, patient's temperature, blood pressure, etc.
- Reduce the on-chip cost of coding in Raspberry Pi 3
- Implementing a mobile application which could be installed on the nurses' phones.

## Figures and Tables

**Table 1:** The advantages and disadvantages of the IV poles existing both in research and in the market.

IV pole types	Method	Advantages	Disadvantages
Research IV poles	Autonomous IV stand	Automated movement	High cost (>2000); complex design; no wireless communication between the nurse and the IV pole; can carry only one IV bag, i.e., can withstand a low weight; consumes a lot of power
	Novel robotic IV pole	Semiautomated movement; saline sensor and alarm; can carry several IV bags; can be manually controlled	Needs patient training; not accurate sensing; no obstacle detection; power consuming

Commercial IV poles	Ambulatory patient support stand	Stable; resembles the walker; helpful for patients with walking difficulties	Occupies a lot of space
	Homecare IV stand	Easy assembly and disassembly; lightweight	Low load capacity, i.e., can hold slight weight merely
	Hanging IV pole	Occupies lower space; low probability of transporting bacteria	Limited mobility area; requires nurse assistance; absence of a place to attach a medical equipment
	Brewer stand	Simple design; high strength; high system stability; smooth movement; most popular	Limited mobility of patients; requires nurse assistance
	Dyaun IV stand	Brake on the wheels	Unstable design

## Data Availability

The data used to support the findings of this study are included within the article.
